# Chronic Effects of Carbamazepine, Progesterone and Their Mixtures at Environmentally Relevant Concentrations on Biochemical Markers of Zebrafish (*Danio rerio*)

**DOI:** 10.3390/antiox11091776

**Published:** 2022-09-08

**Authors:** András Ács, Xinyue Liang, Illés Bock, Jeffrey Griffitts, Bence Ivánovics, Erna Vásárhelyi, Árpád Ferincz, Zsolt Pirger, Béla Urbányi, Zsolt Csenki

**Affiliations:** 1 Department of Freshwater Fish Ecology, Hungarian University of Agriculture and Life Sciences, Páter Károly u. 1., H-2100 Gödöllő, Hungary; 2 Department of Environmental Toxicology, Institute of Aquaculture and Environmental Safety, Hungarian University of Agriculture and Life Sciences, Páter Károly u. 1., H-2100 Gödöllő, Hungary; 3 Balaton Limnological Research Institute, Eötvös Loránd Research Network, Klebelsberg Kuno u. 3, H-8237 Tihany, Hungary; 4 Department of Aquaculture, Hungarian University of Agriculture and Life Sciences, Páter Károly u. 1., H-2100 Gödöllő, Hungary

**Keywords:** carbamazepine, progesterone, oxidative stress, fish biomarker, zebrafish (*Danio rerio*), chronic effects, joint toxicity

## Abstract

The impact of pharmaceuticals on non-target organisms in the environment is of increasing concern and study. Pharmaceuticals and other pollutants are often present as mixtures in an environmental compartment. Studies on the toxicological implications of these drugs on fish, particularly as mixtures at environmentally relevant concentrations, are very limited. Thus, this study aimed to evaluate the chronic effects of the anticonvulsant drug carbamazepine (CBZ) and progesterone (P4) at environmentally relevant concentrations, individually and in binary mixtures, applying a suite of biomarkers at the molecular level in zebrafish (*Danio rerio*). The effects on biotransformation enzymes 7-ethoxyresorufin O-deethylase (EROD) and glutathione-S-transferase (GST), antioxidant enzymes catalase (CAT), superoxide dismutase (SOD), glutathione peroxidases (GPxSe and GPxTOT), and glutathione reductase (GR), and markers of damage, such as DNA strand breaks (DNAsb), lactate dehydrogenase (LDH), lipid peroxidation (LPO), and vitellogenin-like proteins (VTG), were evaluated. Analyses of the biochemical markers indicated that a synergistic dose-ratio-dependent effect of CBZ and P4 in zebrafish occurs after chronic exposure regarding VTG, biotransformation enzymes (EROD, GST), and oxidative stress marker (DNAsb). The results suggest a synergistic effect regarding VTG, thus indicating a high risk to the reproductive success of fish if these pharmaceuticals co-occur.

## 1. Introduction

Pharmaceuticals and their metabolites are found in almost every river worldwide [1]. These pharmaceuticals are released into the environment in high amounts mainly through wastewater treatment plants (WWTP), which have very limited removal or biodegrading efficiency [2,3]. In surface waters, these chemicals may undergo bioaccumulation [4] and/or exert harmful effects in living organisms [5], particularly fish [6,7,8]. Pharmaceuticals are biologically active chemicals, proposed to interact with specific processes and biochemical processes in target species; however, changes in similar metabolic pathways on non-target organisms exposed to pharmaceuticals may also occur [4]. Thus, this pollutant group receives high attention nowadays [9]. 

The anticonvulsant drug, carbamazepine (CBZ), is used worldwide for the treatment of bipolar disorder, trigeminal neuralgia, and psychomotor epilepsy [10]. CBZ is the most frequently detected pharmaceutical in rivers worldwide [1]. Due to its high level of consumption, human healthcare serves as a continuous source of CBZ release to the environment [11] since the majority (72%) of the received amount enters sewage within urine [12]. WWTPs are unable to remove CBZ effectively and only 10% can be removed of the total CBZ amount that enters WWTPs [13]. CBZ is one of the most common pharmaceutical residues found in rivers worldwide [1], and when it is released into the environment within WWTP effluent it is slow to degrade and has been found to have an 82-day permanency time in surface waters [14]. The highest reported concentrations were up to 150 µg/L in South Korea [15]. In Europe, the average amount of CBZ detected was 12 µg/L [16], while in Hungary (river Danube), 0.8 µg/L was detected [17,18].

In humans, CBZ interacts with potassium and sodium channels, as well as some signaling pathways [19]. CBZ is also known to modulate voltage-gated sodium channels, resulting in reduced neuronal activity [20]. Regarding freshwater habitats, despite the high attention CBZ receives, few studies have been performed. The studies that have been conducted in the last decade have utilized algae, cladocerans, and fish [8,21,22,23] to understand the lethal and sublethal effects of CBZ. There is a lack of knowledge concerning the chronic effects of CBZ [24].

In zebrafish (*Danio rerio*), the bioconcentration factor (BCF) of CBZ in plasma was found to be 0.83–1.45, with the half-life for total depuration of tissues being 0.48 ± 0.19 days [25]. Previous zebrafish research has indicated the potential endocrine-disrupting effects of CBZ by noting chronic changes in fecundity, embryo production, and oocytes upon exposure to CBZ [26]. Additional CBZ zebrafish research has found that DNA damage can occur, as well as several enzymes (acetylcholinesterase (AChE), liver glutathione-S-transferase (GST), catalase (CAT), and lactate dehydrogenase (LDH)) are impacted, after 63 days of exposure to CBZ at environmentally relevant (10 µg/L) concentrations [8].

Similar to the zebrafish findings, when common carp (*Cyprinus carpio*) are exposed to environmentally relevant concentrations (1–100 µg/L) of CBZ, there are alterations in several enzymes related to oxidative stress (CAT, SOD, GR, DNAsb), toxicant biotransformation (EROD, GST), and organ and tissue damage (LDH, AChE). In the same study, vitellogenin-like protein levels were found to increase following CBZ exposure, supporting the assumption that CBZ may have endocrine-disrupting effects [27]. Li et al. [28] demonstrated elevated lipid peroxidation in the brain tissues of rainbow trout (*Oncorhynchus mykiss*) accompanied by inhibition in the activity of SOD and GR, with glutathione peroxidase and CAT showing a non-linear response in the function of time, with an increase followed by a reduction in their activities after CBZ exposure.

Natural progesterone (4-Pregnene-3,20-dione, P4) is generally used in combination with estrogens as an oral contraceptive and in hormone replacement therapy. As a result of their extensive usage combined with their excretion within human and animal feces and urine into surface waters through WWTPs, progestogens are often detected in a concentration range of 0.07 to 22.2 ng/L [9,29]. P4 is stable in sterile water but undergoes biodegradation in the presence of some algae strains [30]. Steroid hormones are considered strong endocrine disruptors [29], of which progestins are the least studied. A few studies have confirmed the adverse effects of progestins on the reproduction and fertility of some freshwater species [31,32,33,34,35].

In zebrafish, the reproductive effect of P4 [36] and synthetic progestins [37] was demonstrated. The BCF of different progestins ranges from 7 (Dienogest) to 128 (Medroxyprogesterone acetate) [38]. More recently, Liang et al. [34] showed that environmentally relevant concentrations of progesterone may affect sex differentiation of zebrafish. Cardoso et al. [33] showed that the synthetic progestin, levonorgestrel, affected the liver and reduced vitellogenin production in female zebrafish, but had no significant effects on CYP1A levels at an environmentally relevant (10 ng/L) concentration after 21 days of exposure. 

Pharmaceuticals and other micro- and macro-pollutants often occur as multi-component mixtures in an environmental compartment [35,39,40,41]. Until now, studies on the toxic effects of these drugs on non-target organisms, such as fish, particularly as mixtures at environmentally relevant concentrations, have been very limited. Moreover, the joint toxic effect of mixtures is typically higher than the toxicity of the compounds individually [42,43]. Even binary mixtures of different compounds often show a similar effect [44,45]. In the last decade, it has become evident that standard acute toxicity tests do not have the adequate sensitivity to assess the effects of pharmaceuticals on aquatic biota as teratogenicity was detected on sea urchin (*Paracentrotus lividus*) after exposure to environmental concentrations of carbamazepine and ibuprofen at a concentration of 0.00001 mg/L [46]. Thus, it is required to use more sensitive response endpoints in toxicology studies, such as biochemical markers at the molecular level.

As CBZ and P4 often co-occur in the environment, this study aims to evaluate their chronic toxicological effects in zebrafish (*Danio rerio*) at environmentally relevant concentrations of these compounds, individually and in binary mixtures. To determine the toxicological effects, we focused on a suite of molecular biomarkers which are widely applied in pharmaceutical toxicity studies with fish and include: the biotransformation enzymes (7-ethoxyresorufin O-deethylase (EROD), glutathione-S-transferase (GST)), antioxidant enzymes (catalase (CAT), superoxide dismutase (SOD), glutathione peroxidases (GPxSe and GPxTOT), glutathione reductase (GR)), and markers of damage (DNA strand breaks (DNAsb), lactate dehydrogenase (LDH), lipid peroxidation (LPO), and vitellogenin-like proteins (VTG)) [27,33,34,47,48]. These biomarkers were selected to screen for and identify any mechanistic effects of oxidative stress and damage, alterations in xenobiotic metabolism, nervous effects, and endocrine disruption.

## 2. Materials and Methods

### 2.1. Chemicals

Carbamazepine (Cbz) (≥100%, CAS 298-46-4) and progesterone (≥99%, CAS 57-83-0) were purchased from Sigma-Aldrich (Darmstadt, Germany). All other reagents were analytical grade.

### 2.2. Test Organisms

The zebrafish (AB wildtype) used in this study were supplied by the zebrafish facility of the Department of Environmental Toxicology at the Hungarian University of Agriculture and Life Sciences (Gödöllő, Hungary). Fish were maintained at constant water quality parameters (25 ± 0.5 °C; pH 7.0 ± 0.2; conductivity 500 ± 50 μS; alkalinity < MDL, 0 mM CO_3_^2−^, 0.4 mM HCO_3_^2−^; hardness < 0.5° dH; DOC > 90%; system water) in a Tecniplast ZebTec (Buguggiate, Italy) recirculating zebrafish housing system. The photoperiod was set to a 14 h light/10 h dark cycle. The fish were fed twice a day with ZEBRAFEED (Sparos, 400–600 µm) and twice a week with brine shrimp (Ocean Nutrition > 230,000 NPG).

### 2.3. Experimental Design

For the subacute, 28-day adult exposure tests, 9–12-month-old male and female fish were randomly distributed into 15 experimental tanks, each containing 3 L of the test solution (nominal concentrations: 0, 1, 5, 50, 100 μg/L of CBZ, or 0, 1, 5, 50, 100 ng/L of P4). Three replicates (with fifteen fish each) were used per treatment. The lowest and highest CBZ concentrations tested, 1 and 100 μg/L, respectively, were selected based on the recent papers by da Silva Santos (CBZ) [8] and Liang (P4) [34]. To evaluate the non-linear response produced by the joint effect of CBZ and P4, the EC_50_ was calculated based on VTG results. VTG was selected due to the expected effect of the test chemicals. Based on toxicity units (TU; 1 TU = concentration of a compound in the mixture per the compound’s EC_50_), mixtures were composed to equal 1 TU (CBZ:P4 ratios were: 0.75 TU:0.25 TU (MIX1), 0.5 TU:0.5 TU (MIX2), and 0.25 TU:0.75 TU (MIX3)) (Table 1). Mixtures were tested in the same setup as described previously (0, MIX1, MIX2, MIX3, 3 replicates, 15 fish each). In all mixtures, the theoretical toxic effect was expected to be 50%. In this setup, non-linear mixture effects (synergistic or antagonistic) were easily identifiable, and the effect of different concentration ratios were observable [49]. Fish were exposed for 28 days and fed once daily with a quantity of ZEBRAFEED (Sparos, 400–600 µm) corresponding to 2% of the fish weight in the aquarium. The exposure media was completely renewed every three days. Water quality parameters were kept within the ranges described in the preceding “Test Organisms” section. Water samples were analyzed during the experimental period by LC-MS/MS to ensure that nominal and actual test compound concentrations in the aquaria were identical. Samples from the aquaria were collected from the test medium after 1 and 36 h of renewing the test solutions. The mean concentrations of CBZ and P4 in the water samples were consistently within 20% of the intended concentrations.

At the 7th, 14th, and 28th days of exposure, 5 fish from each exposure concentration and replicate were sacrificed after an anesthetic overdose (0.04% MS-222 (tricaine-methane-sulphonate) (Sigma-Aldrich, Darmstadt, Germany)). The brain, liver, and intestine of each fish were isolated and stored in microtubes at −80 °C for later biochemical analyses.

### 2.4. Biochemical Determinations

Homogenization and biochemical determinations were performed as described by Liang et al. [27]. More detailed procedures are described in Supplement S1.

### 2.5. Statistical Analysis

All data were analyzed as described by Liang et al. [27], with the exception of categorical predictor factors, which was the treatment (control, 1, 5, 50, 100 µg/L, 1, 5, 50, 100 ng/L, or MIX1, MIX2, MIX3) in this study.

## 3. Results

No fish died, and no visible sublethal effects were detected during the experimental assay (control, CBZ-, P4-, or mixture-exposed) at any of the tested conditions.

### 3.1. Vitellogenin-Like Proteins

**Figure 1:**
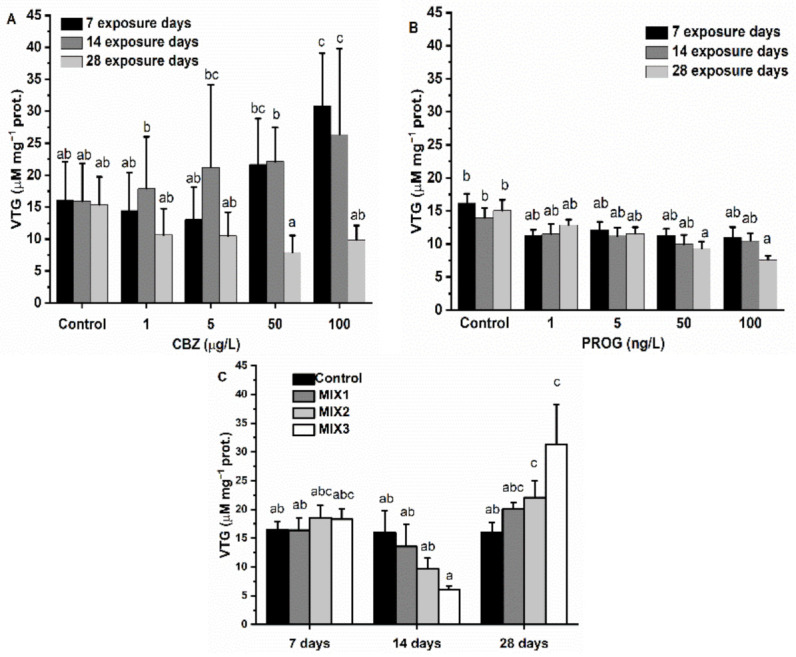
Changes in VTG content in the gonads of zebrafish exposed to (**A**) CBZ, (**B**) P4, and (**C**) binary mixtures of CBZ and P4 for 7, 14, and 28 days. Data are expressed as mean ± standard deviation of fifteen replicates (*n* = 15). Different letters indicate significant differences at *p* < 0.05 after a two-way ANOVA followed by Tukey’s post hoc test.

After the first and second weeks of exposure to CBZ, VTG levels increased following a concentration-dependent pattern, however, a significant (*p* < 0.05) elevation of VTG content in the samples was detected only in fish exposed to 100 µg/L of CBZ. After 28 days, VTG levels followed a concentration-dependent pattern, but this decrease was not significant (*p* < 0.05) at any exposure concentration (Figure 1A). P4 caused a decrease in the VTG content of fish samples after exposure to all applied concentrations (1, 5, 50, 100 ng/L). Concentration and time dependence were also confirmed by statistics; however, significant differences in VTG levels were only observable in fish exposed to 50 and 100 ng/L of P4 for 28 days (Figure 1B). After seven days of exposure to MIX1, MIX2, or MIX3, no significant change in VTG levels was visible. Two weeks of exposure to mixtures caused a non-significant (*p* < 0.05) drop in VTG levels, and VTG concentrations decreased with growing proportions of P4 in mixtures compared to control group levels. After 28 days of exposure to mixtures, VTG levels followed an increasing pattern in proportion to P4 and reached a significant (*p* < 0.05) increase in the case of MIX2 and MIX3 (Figure 1C).

### 3.2. AChE Activity

**Figure 2:**
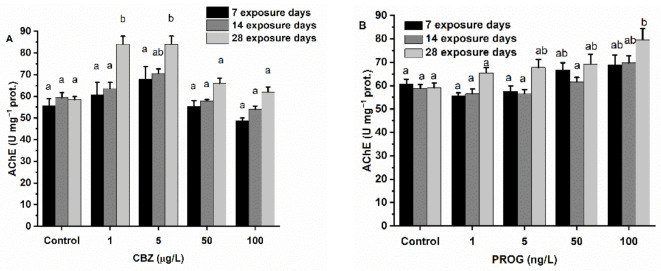

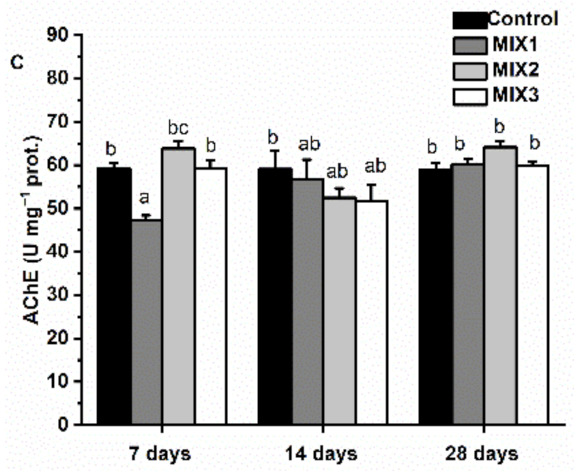
Changes in AChE activity in the brain of *Danio rerio* exposed to (**A**) CBZ, (**B**) P4, and (**C**) binary mixtures of CBZ and P4 for 7, 14, and 28 days. Data are expressed as mean ± standard deviation of fifteen replicates (n = 15). Different letters indicate significant differences at *p* < 0.05 after a two-way ANOVA followed by Tukey’s post hoc test.

The results of AChE activity measured in the brain of zebrafish after exposure to CBZ, P4, and mixtures containing both chemicals in different proportions are shown in Figure 2A–C. After seven days of exposure to CBZ, no significant change was observable in AChE activity, although an initial increase, peaking at a 5 µg/L CBZ concentration, followed by a drop in AChE activity, was visible. This pattern was also apparent after 14 and 28 days of exposure to CBZ. A significant increase was observable after 14 days of 5 µg/L and 28 days of 1 and 5 µg/L of CBZ exposure. P4 induced an increase in AChE activity, showing significant (*p* < 0.05) time and concentration dependence. After the first and second weeks of exposure to 50 and 100 ng/L, P4 caused significantly higher AChE activity compared to activity values measured in control groups. After 28 days, AChE significantly (*p* < 0.05) increased at concentrations above 5 ng/L (5, 50, 100 ng/L) of P4. After 7 days of exposure to mixtures of CBZ and P4, a significant (*p* < 0.05) drop was observable in the case of MIX1. MIX2 and MIX3 did not cause any significant change in AChE activity compared to control group values. After 14 and 28 days, exposure to MIX1, MIX2, and MIX3 resulted in no significant change in AChE activity (Figure 2).

### 3.3. Biotransformation Enzymes

**Figure 3:**
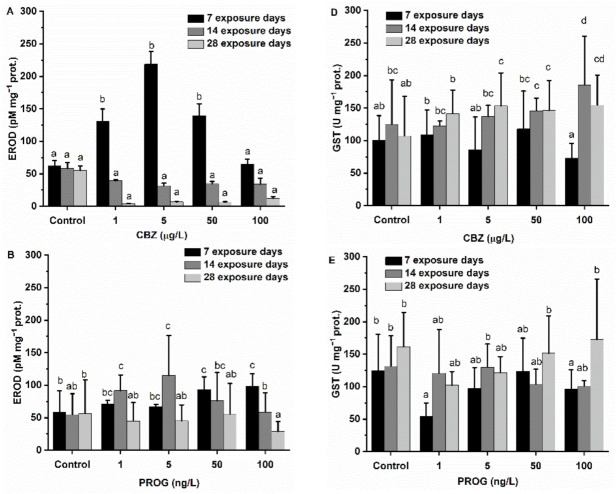

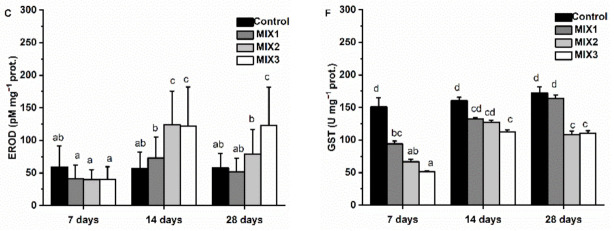
Changes of biotransformation enzymes’ activity—hepatic EROD (**A**–**C**) and GST (**D**–**F**) in the liver of *Danio rerio* exposed to (**A**,**D**) CBZ, (**B**,**E**) P4, and (**C**,**F**) binary mixtures of CBZ and P4 for 7, 14, and 28 days. Data are expressed as mean ± standard deviation of fifteen replicates (n = 15). Different letters show significant differences at *p* < 0.05 after a two-way ANOVA followed by Tukey’s post hoc test.

After 7 days of exposure to 1, 5, and 50 µg/L of CBZ, hepatic EROD activity levels in fish showed a significant (*p* < 0.05) increase compared to the control group. The highest EROD activity measured was observed at 5 µg/L of CBZ. Interestingly, 100 µg/L of CBZ had no significant effect on EROD activity. After 14 and 28 days of CBZ exposure, a non-significant decrease in EROD activity was detected as compared to the controls and between different CBZ concentration exposures. P4 induced EROD activity in a concentration-dependent way: significantly (*p* < 0.05) higher activity was measured at 50 and 100 ng/L P4 concentrations as compared to activity levels measured in the control group. After two weeks of exposure to P4, EROD levels increased significantly (*p* < 0.05) at lower concentrations of P4 (1, 5 ng/L), which was followed by the inhibition of EROD activity at the 100 ng/L P4 concentration. After four weeks of P4 exposure, EROD activity was decreased at all exposure concentrations, with EROD inhibition being significant at the 100 ng/L P4 concentration. After the first week, CBZ and P4 mixtures caused a non-significant decrease in EROD activity. After two weeks of exposure to MIX1, MIX2, and MIX3, hepatic EROD levels showed an increased activity, with a significant difference being observed in MIX2 and MIX3 as compared to control group activity levels. After 28 days, MIX1 did not alter EROD activity, but MIX2 and MIX3 still induced EROD activity, with MIX3 being significantly higher as compared to control activity levels (Figure 3A–C).

GST activity at different exposure concentrations did not cause a significant change after the first week of CBZ exposure. After 14 and 28 days, GST levels showed a concentration-dependent increase: significantly increased activities were observed after 14 days of exposure to 100 µg/L, and after 28 days of exposure to 5, 50, and 100 µg/L of CBZ. Significant (*p* < 0.05) P4-induced concentration-dependent GST activity changes were detectable only after 28 days at 50 and 100 ng/L P4 concentrations. A significant decrease in GST activity was found at 1 and 100 ng/L of P4 exposure after one week, but other test concentrations did not trigger any change after one or two weeks. The CBZ and P4 mixtures (MIX1, MIX2, MIX3) caused a significant (*p* < 0.05) decrease in GST activity in a concentration-dependent manner. An increasing inhibitory effect was observed in proportion to the amount of P4 (MIX1 < MIX2 < MIX3) present in the mixtures. The inhibitory effect of the mixtures was still detectable after two and four weeks of exposure, but differences as compared to the control group were not so pronounced; specifically, a significant inhibition was only found in MIX2 after two weeks, and after four weeks in MIX2 and MIX3 (Figure 3C–E).

### 3.4. Antioxidant Defense System

**Figure 4:**
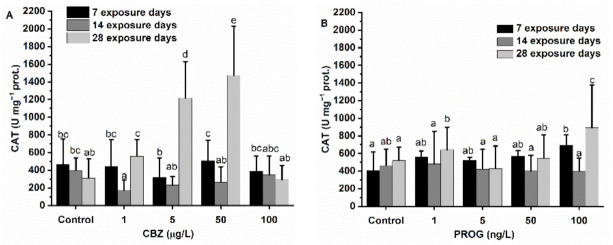

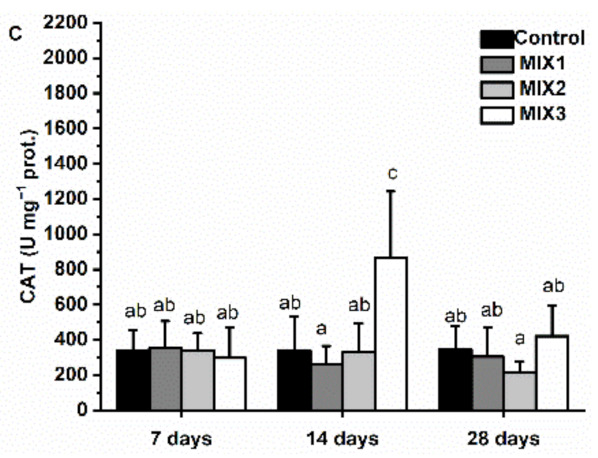
Changes in CAT activity in the liver of *Danio rerio* exposed to (**A**) CBZ, (**B**) P4, and (**C**) binary mixtures of CBZ and P4 for 7, 14, and 28 days. Data are expressed as mean ± standard deviation of fifteen replicates (n = 15). Different letters indicate significant differences at *p* < 0.05 after a two-way ANOVA followed by Tukey’s post hoc test.

CBZ did not induce any concentration-dependent changes in CAT activity until the fourth week of exposure. A significant change (decrease) was only observed at the 1 µg/L CBZ concentration after two weeks. After 28 days, a concentration-dependent increase was observed in the 1, 5, and 50 µg/L CBZ exposure groups. At the 100 µg/L CBZ concentration, no significant difference in CAT activity was detected as compared to the levels measured in the control group. P4 significantly (*p* < 0.05) altered CAT activity after exposure to 100 ng/L of P4 for four weeks as compared to the control groups. Other CAT activity changes remained non-significant, with no time nor concentration dependency being supported by statistics. In the binary mixtures of CBZ and P4, only MIX3 triggered a significant increase in CAT activity after two weeks of exposure (Figure 4).

**Figure 5:**
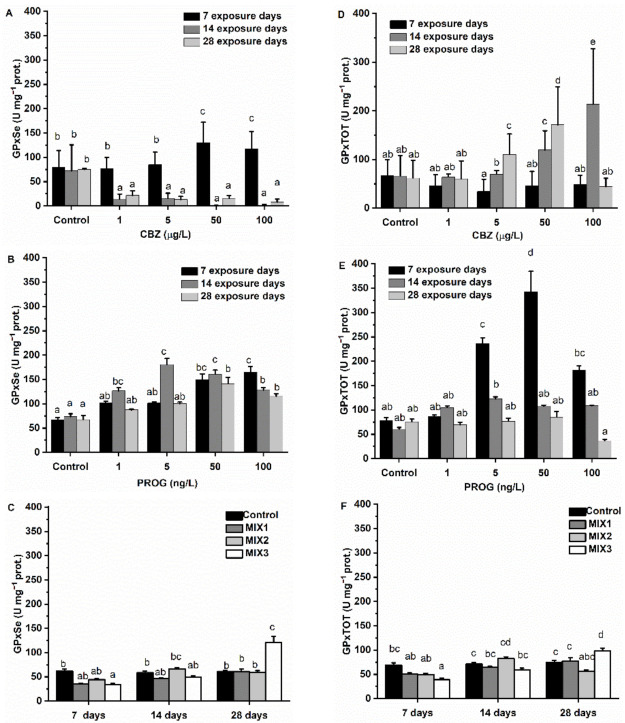
Changes of biotransformation enzymes’ activity—GPxSe (**A**–**C**) and GPxTOT (**D**–**F**) in the liver of *Danio rerio* exposed to (**A**,**D**) CBZ, (**B**,**E**) P4, and (**C**,**F**) binary mixtures of CBZ and P4 for 7, 14, and 28 days. Data are expressed as mean ± standard deviation of fifteen replicates (n = 15). Different letters indicate significant differences at *p* < 0.05 after a two-way ANOVA followed by Tukey’s post hoc test.

GPxSe was increased after the first week of exposure to CBZ. The increase was concentration-dependent and significant (*p* < 0.05) at 50 and 100 µg/L CBZ concentrations. After 14 and 28 days of exposure to CBZ, at all applied concentrations, GPxSe was strongly decreased in a time- and concentration-dependent manner, with the decrease being significant (*p* < 0.05) at all exposure concentrations. Exposure to CBZ did not affect GPxTOT activity after seven days of exposure. GPxTOT increased significantly (*p* < 0.05) after two weeks at exposure concentrations of 5, 50, and 100 µg/L of CBZ. After 28 days of exposure, 5 and 50 µg/L of CBZ exposure significantly (*p* < 0.05) increased the activity of GPxTOT in zebrafish, but at the 100 µg/L CBZ concentration, GPxTOT activity was not different when compared to the activity levels of the control group. After exposure to P4, GPxSe was increased in a time- and concentration-dependent manner at all applied concentrations and time points. After the first week of P4 exposure, GPxSe increased significantly (*p* < 0.05) at 50 and 100 ng/L of P4. After two weeks of exposure, the increase reached a significant level at exposure concentrations of 1, 5, 50, and 100 ng/L of P4. There was a peak activity detectable at the 5 ng/L concentration and the extent of the increase dropped slightly at the 50 and 100 ng/L P4 concentrations. This pattern was also observable after 28 exposure days. GPxSe activity levels peaked at 50 ng/L of P4, with a drop being observed at the 100 ng/L exposure concentration. The results of GPxTOT activity showed an initial increase in activity, peaking at 50 ng/L of P4 after one week of exposure, followed by a slight decrease. A significant difference (*p* < 0.05) was confirmed at 5, 50, and 100 ng/L P4 concentrations. This pattern was also observed after the second exposure week: GPxTOT activity peaked at 5 ng/L of P4 and decreased slightly afterwards (50, 100 ng/L). Significance (*p* < 0.05) was confirmed in the 5 ng/L P4 concentration only. After the fourth exposure week, a significant (*p* < 0.05) alteration (decrease) of GPxTOT activity was only detected in the 100 ng/L P4 concentration (Figure 5).

Binary mixtures caused significant (*p* < 0.05) changes in both GPxSe and GPxTOT activity in MIX3 after one week of exposure (decrease), and after four weeks of exposure (increase).

**Figure 6:**
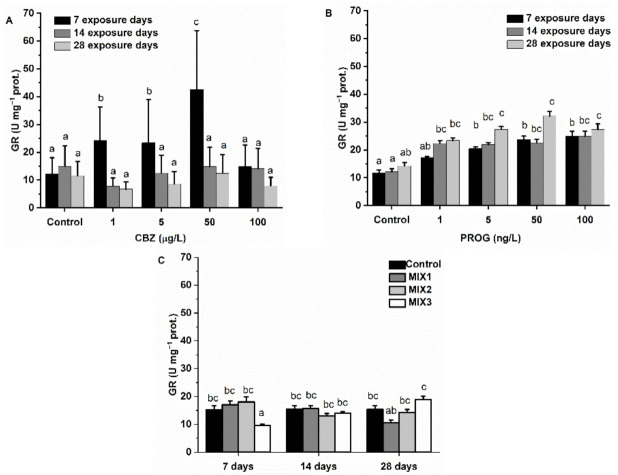
Changes in GR activity in the liver of *Danio rerio* exposed to (**A**) CBZ, (**B**) P4, and (**C**) binary mixtures of CBZ and P4 for 7, 14, and 28 days. Data are expressed as mean ± standard deviation of fifteen replicates (n = 15). Different letters show significant differences at *p* < 0.05 after a two-way ANOVA followed by Tukey’s post hoc test.

GR activity was significantly increased after the first week in fish exposed to 1, 5, and 50 µg/L of CBZ, but at the 100 µg/L CBZ concentration, no significant difference was found as compared to control group activity values. Additionally, at a longer timescale (14 and 28 days), no significant change in GR activity was detected, however, a time-dependent tendency was supported by statistics. In response to P4, GR activity increased significantly (*p* < 0.05) at exposure concentrations above 1 ng/L (5, 50, 100 µg/L) in a time- and concentration-dependent manner, which was supported by statistics. Regarding binary mixtures, only MIX3, after seven days of exposure, decreased the activity of GR significantly (*p* < 0.05). No other significant effects were observable during the 28 days of exposure (Figure 6).

**Figure 7:**
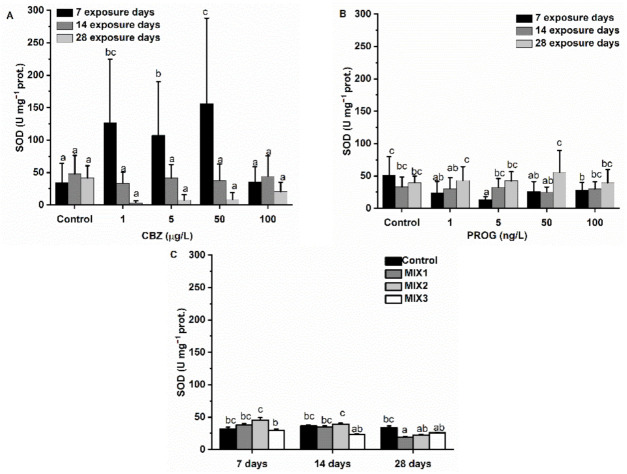
Changes in SOD activity in the liver of *Danio rerio* exposed to (**A**) CBZ, (**B**) P4, and (**C**) binary mixtures of CBZ and P4 for 7, 14, and 28 days. Data are expressed as mean ± standard deviation of fifteen replicates (*n* = 15). Different letters show significant differences at *p* < 0.05 after a two-way ANOVA followed by Tukey’s post hoc test.

CBZ induced a strong significant (*p* < 0.05) increase in SOD activity after seven days of exposure at 1, 5, and 50 µg/L CBZ concentrations when compared to control group values. At the highest CBZ concentration (100 µg/L), SOD activity fell in relation to control values. After the second and fourth exposure weeks, SOD values were significantly (*p* < 0.05) lower as compared to control values in all exposure concentrations (1, 5, 50, and 100 µg/L). Time and concentration dependency were also confirmed by statistics. Compared to control values, P4 significantly (*p* < 0.05) altered (decreased) SOD activity only at the 5 ng/L concentration after one week of exposure. Other changes were not significant, and no concentration or time dependency was supported by statistics. The binary mixtures significantly (*p* < 0.05) reduced the SOD activity only in MIX1 after 28 days of exposure (Figure 7).

### 3.5. Damage Markers

**Figure 8:**
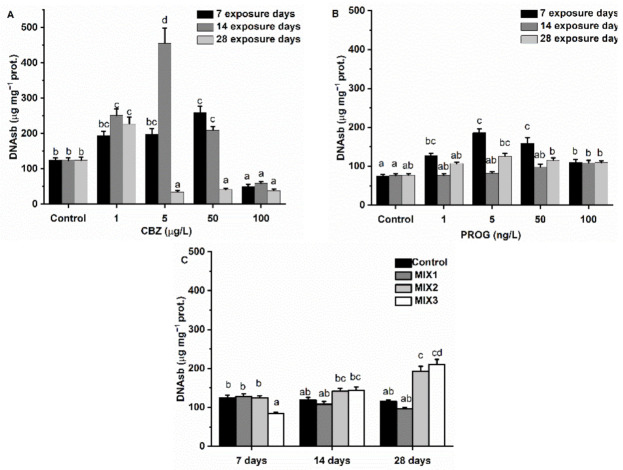
Changes in DNAsb concentration in the liver of *Danio rerio* exposed to (**A**) CBZ, (**B**) P4, and (**C**) binary mixtures of CBZ and P4 for 7, 14, and 28 days. Data are expressed as mean ± standard deviation of fifteen replicates (n = 15). Different letters show differences at *p* < 0.05 after a two-way ANOVA followed by Tukey’s post hoc test.

After one week, DNAsb values showed an increase after 1, 5, and 50 µg/L of CBZ exposure, with a significant (*p* < 0.05) increase at 50 µg/L. At a concentration of 100 µg/L of CBZ, no significant difference was measured as compared to control values. Two weeks of exposure caused significantly (*p* < 0.05) increased DNAsb values at concentrations of 1, 5, and 50 µg/L of CBZ, with the highest levels appearing at 5 µg/L of CBZ. After 28 days and only at the 1 µg/L CBZ concentration, elevated DNAsb levels were significant (*p* < 0.05). At 5, 50, and 100 µg/L of CBZ, a significant decrease was observed. P4 caused a significant elevation of DNAsb after one week of exposure at concentrations of 1, 5, 50, and 100 ng/L. The highest DNAsb values were measured in 5 ng/L of P4-exposed samples. After two weeks of exposure, 50 and 100 ng/L of P4 significantly increased the DNAsb content of the samples. By the fourth week of exposure, none of the applied exposure concentrations of P4 caused any significant changes in DNAsb concentrations. After exposure to binary mixtures for one week, only MIX3 significantly (*p* < 0.05) altered DNAsb (decreased) values as compared to controls. After 28 days, MIX2 and MIX3 significantly increased the measured DNAsb values (Figure 8).

**Figure 9:**
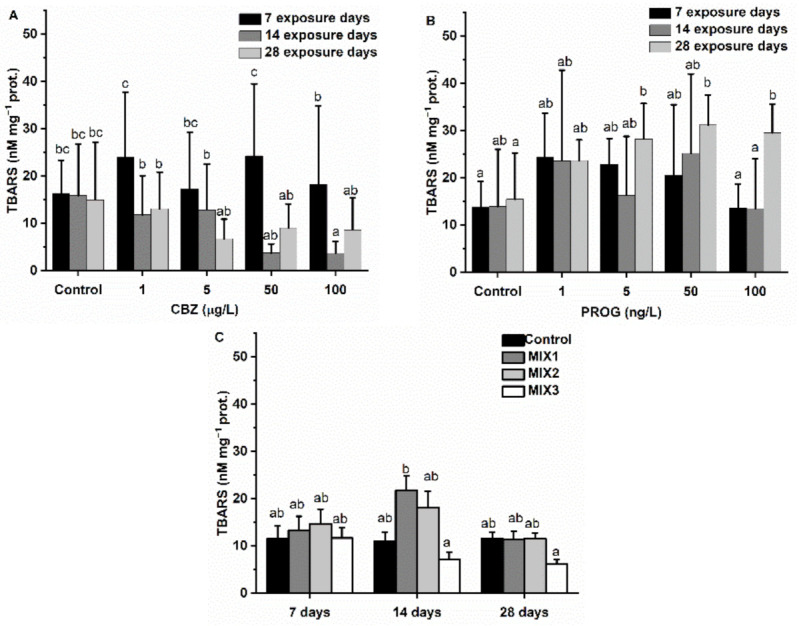
Changes in LPO concentration in the liver of *Danio rerio* exposed to (**A**) CBZ, (**B**) P4, and (**C**) binary mixtures of CBZ and P4 for 7, 14, and 28 days. Data are expressed as mean ± standard deviation of fifteen replicates (*n* = 15). Different letters show significant differences at *p* < 0.05 after a two-way ANOVA followed by Tukey’s post hoc test.

CBZ significantly (*p* < 0.05) altered (decreased) the LPO content of the samples at a concentration of 100 µg/L of CBZ as compared to control samples. Other changes were not significant during the exposure time of four weeks. A decreasing tendency was observable in LPO content after two and four weeks of CBZ exposure. Compared to control values, P4 increased the LPO content in samples exposed to 5, 50, and 100 ng/L concentrations for four weeks. Binary mixtures did not significantly affect LPO content in zebrafish during the four-week exposure time (Figure 9).

**Figure 10:**
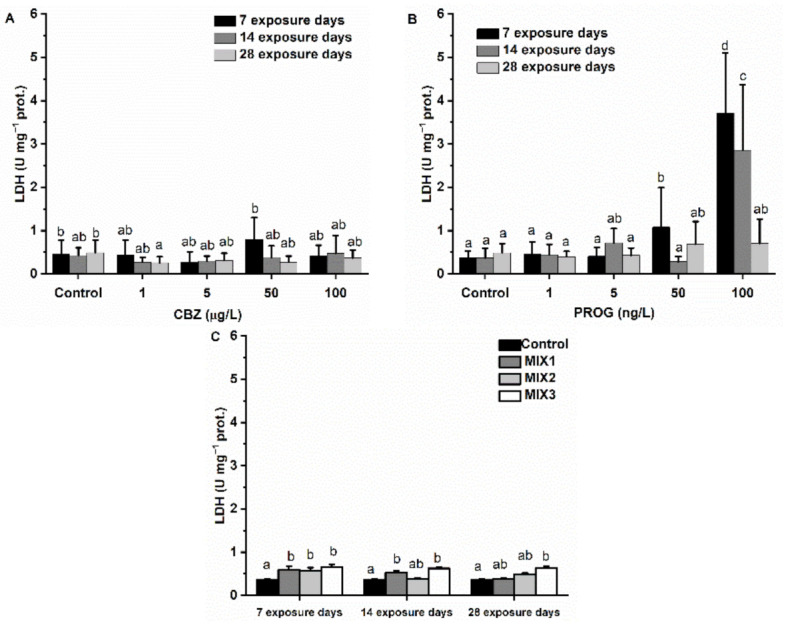
Changes in LDH activity in the liver of *Danio rerio* exposed to (**A**) CBZ, **(B**) P4, and (**C**) binary mixtures of CBZ and P4 for 7, 14, and 28 days. Data are expressed as mean ± standard deviation of fifteen replicates (*n* = 15). Different letters indicate significant differences at *p* < 0.05 after a two-way ANOVA followed by Tukey’s post hoc test.

CBZ did not affect LDH activity during the four-week exposure. P4 significantly (*p* < 0.05) increased LDH activity in samples after one week of exposure to 50 and 100 ng/L, and after two weeks of 100 ng/L. MIX1, MIX2, and MIX3 significantly increased the LDH activity after one week. After two weeks of exposure to MIX2 and MIX3, there were significantly altered LDH levels. MIX3 also increased activity levels after 28 days of exposure (Figure 10).

## 4. Discussion

In recent studies, CBZ was shown to have a negative effect on the reproductive success of zebrafish and was suggested to have similar toxic routes as other estrogenic compounds [8]. Fish VTG is a glycolipophosphoprotein produced in the liver of fish, and its production increases in response to 17β-estradiol or compounds which are capable of interacting with the estrogen receptor, increasing estrogens and decreasing androgens [50,51,52,53]. CBZ was shown to induce VTG production in common carp (*Cyprinus carpio*) subjected to 100 µg/L of CBZ for seven days. Correspondingly, in this study, the VTG concentration in zebrafish was increased following CBZ exposure for one and two weeks, with a significant elevation being detected in fish subjected to the 100 µg/L concentration. Conversely, VTG levels decreased slightly after 28 days, as compared to the values in control groups. P4 and other synthetic progestins are known to alter VTG levels in fish, with the inhibition or induction of VTG production being dependent on the type of progestogen and the applied concentrations, if applied in combination [54,55]. In the present study, a time- and concentration-dependent decrease in VTG concertation was demonstrated. A significant drop in VTG was observed after 28 days of exposure to 50 and 100 ng/L of P4. Binary mixtures of CBZ and P4 significantly altered VTG production after 28 days and VTG production was increased after exposure to MIX1 and MIX2, but MIX1 had no significant effect. After two weeks, a non-significant decrease in VTG content was observed following an increasing proportion of P4 and a decrease in CBZ concentration. An initial drop, or increase, in VTG concentration may be attributed to a hormetic effect, which often appears in endocrine signaling [56]. Increasing VTG concentrations in fish after long-term exposure to mixtures of CBZ and P4 may support the suggestion that CBZ and P4 are acting together as synergic compounds. The increase in the proportion of P4 seemed to increase and prolong the CBZ effect at the tested concentration range.

The increasing AChE activity after CBZ exposure found in this study is in agreement with other zebrafish studies [8]. In this study, only 1 and 5 µg/L CBZ concentrations caused a significant increase after 28 days, with time and concertation dependency being supported by statistics. The decrease at higher exposure concentrations may be from other toxic effects. After 28 days, P4 also increased the level of AChE activity at the 100 ng/L exposure concentration. Previous animal studies have shown a relationship between increased AChE activity, oxidative stress [57], the production of free radicals, and apoptotic processes [58,59]. Physiologically, AChE breaks down the neurotransmitter acetylcholine, resulting in decreased acetylcholine receptor stimulation and affecting an organism’s cognitive function [60].

The phase I and phase II biotransformation enzymes EROD and GST are commonly used in fish biomarker screening to detect the uptake and metabolism of environmental organic pollutants. EROD is regulated by the aryl hydrocarbon (AhR) receptor and is a member of the P450-dependent monooxygenase CYP1A family [61,62]. After seven days of exposure to CBZ, increasing EROD activity shows that CYP1A enzymes were biosynthesized to detoxify and metabolize CBZ. This result agrees with the findings of a previous study with *Carassius carassius*, where 2 and 10 µg/L CBZ concentrations were proven to elevate hepatic EROD activity after 1, 4, and 7 days [48]. The subsequent decrease after 14 and 28 days of exposure to CBZ may be attributed to adaptations to the chemical stressor or changing metabolism of CBZ. Initially, EROD activity was significantly (50, 100 ng/L) increased after the first and second weeks of exposure to P4, then later significantly decreased by the 28th exposure day. None of the assessed mixtures caused a significant effect in EROD activity after 1 week of exposure, but MIX2 and MIX3 caused a significant increase after 14 and 28 days. GST did not show increased activity after the first seven days of CBZ exposure, then it increased significantly after the second and remained significantly elevated during the fourth exposure week. In previous studies, GST was also shown to increase in *Cyprinus carpio*, *Carassius carassius,* and *Danio rerio* after exposure to environmentally relevant concentrations (1–100 µ/L) of CBZ [8,27,48]. Regarding the effect of P4 on GST, only the lowest applied exposure concentration (1 ng/L) triggered a significant change in GST activity, most probably due to a hormetic response. The observed pattern of the mixtures in GST and EROD activity may suggest an altered metabolic route for xenobiotics. In the case of EROD activity, mixtures of CBZ and P4 seemed to shift significant effects in time, mitigating short-term effects and causing a significant increase in chronic effects. Chronic effects were absent in single-compound exposures. For GST, the observed effect was increasing in relation to a growing P4 ratio (MIX1 < MIX2 < MIX3). These observations suggest that mixtures of P4 and CBZ may have a synergistic effect on a chronic timescale, becoming more pronounced with the proportion of P4.

In fish, phase I metabolism (e.g., EROD) produces reactive oxygen species (ROS) as by-products [63]. Cells protect themselves against ROS, including non-enzymatic scavengers (e.g., reduced glutathione, GSH) and antioxidant enzymes. The most abundant antioxidant enzymes, such as CAT, SOD, and GPx, are found in the peroxisomes of fish liver cells [64,65,66]. SOD is the first line of antioxidant defense against ROS, and second-line enzymes such as CAT and selenic-dependent GPx, which break down H_2_O_2_. GR converts oxidized glutathione (GSSG) back to its reduced form (GSH) if oxidized by ROS, playing a key role in glutathione balance [67,68]. In this study, CBZ stimulated SOD, GR, and GPxSe activity after the first exposure week, resulting in a significantly high activity of SOD, GR, and GPxSe. Subsequently, SOD and GR activities dropped to control levels at 100 µg/L, even at the first week, and following the second week, they remained slightly suppressed until the 28th day of exposure, as seen by EROD activities. Conversely, GPxTOT and CAT activities were not altered until the second exposure week. After the second week, GPxTOT, and then after the fourth week both CAT and GPxTOT activities were increased significantly at 5 and 50 µg/L CBZ concentrations. After exposure to 100 µg/L of CBZ for 28 days, GPxTOT and CAT activities decreased to control levels. The first week results suggest that antioxidant enzyme activities were increased as a consequence of inorganic ROS produced by EROD (or other phase I metabolites) and/or by SOD activity, which were neutralized by GPxSe and GSH (as indicated by increased GR activity). The decreasing GST activity also reflects the diminishing amount of glutathione after the first week. After the second exposure week, most probably, EROD and SOD were failing to perform their functions, and organic ROS were becoming predominant. There is evidence that antioxidant enzyme activities may decrease under excess ROS production, if, for example, superoxide radicals not eliminated by SOD are able to inhibit CAT or GPxSe, and proteins inhibiting other antioxidant enzymatic activities [69,70]. It is also important to note that CAT and GPxSe have complementary roles in H_2_O_2_ elimination [70], with each having different subcellular localizations, such as peroxisomal (GPx) versus mitochondrial and cytosolic fractions (CAT) [63], as well as different target molecules (reduction of H_2_O_2_ by CAT and GPxSe, while selenic-independent GPx is able to reduce toxic hydroperoxides) [71]. In the present case, a failure of phase I metabolism, including EROD which should eliminate organic xenobiotics, may have led to excess ROS, and thus resulted in the observed increased GPxTOT and CAT activity, and the inhibition of SOD, GPxSe, and GR. It is also plausible that an energy (NADPH) shortage following exposure to CBZ was causing the observed effects [72,73].

Antioxidant system enzymes’ results of P4-exposed fish, meant to neutralize inorganic ROS, were following the pattern of EROD activity changes: GR and GPxSe were increased significantly after one week of exposure (50 and 100 ng/L of P4), and remained significantly higher than control values during the second (1, 5, 50, 100 ng/L of P4) and fourth weeks (1, 5, 50, 100 ng/L of P4). CAT showed significantly elevated activity at the 100 ng/L P4 concentration after 28 days. SOD was not affected by P4 exposure. These results also suggest that phase I metabolism or other processes producing inorganic ROS were mainly causing the measured enzyme activity changes.

Regarding the binary mixtures of CBZ and P4, MIX3 caused significant alterations of antioxidant enzymes GR, GPxSe, and GPxTOT. After the first exposure week, an inhibition in the activity of GPxSe, GPxTOT, and GR was observed. At the fourth week, GPxSe and GPxTOT showed significantly higher activity as compared to control group values. After 28 days, MIX1 caused a significant decrease in SOD activity. This result may suggest that the metabolism or mode of action of the mixture of these compounds may differ from the single chemical’s effect, as also suggested by xenobiotic metabolization enzymes’ results, and the mode of action of the antioxidant system depends not only on exposure time, but also on the proportion of CBZ and P4. Mixtures seem to mitigate, or even reverse, the short- and long-term effects of all assessed antioxidant enzymes predicted from the single-solution results. It is notable that MIX3, containing the highest proportion of P4, had the most significant effects.

Oxidative stress is known to cause damage to cellular lipids, proteins, and DNA [74]. A common indicator of oxidate damage to lipids, lipid peroxidation (LPO), is TBARS [75]. CBZ exposure did not cause elevated LPO levels of zebrafish in this study. After four weeks of exposure to P4, TBARS levels were found to be significantly higher at 5, 50, and 100 ng/L concentrations, supporting the finding that exogenic P4 causes oxidative stress in zebrafish. Binary mixtures did not cause increased TBARS levels in zebrafish during our assessments. However, reduced LPO levels were observed after two and four weeks of exposure to MIX3. The reduction in TBARS levels may be attributed to the lower lipid content of the cells [76], suggesting a deteriorating condition within the fish. Initially, increased DNAsb levels after CBZ exposure may be attributed to oxidative damage of the genetic material of the cells following oxidative stress, as suggested by ROS defense enzyme results. Subsequent observations of low DNAsb levels may be a result of the initiation of repair and recovery mechanisms [77] initiated by oxidative stress effects, and/or the inhibition of cell division [78]. In the P4 treatment, DNAsb levels were significantly higher as compared to control groups after one week of exposure. After two and four exposure weeks, DNAsb levels were found to not be significantly different from control group levels, suggesting that the antioxidant system may cope with the oxidative stress. The decrease after one week of exposure to MIX1 and the significantly higher levels of DNAsb after four weeks of exposure to MIX2 and MIX3 are suggestive of an initial activation of the DNA repair mechanism at the first week and genetical material being damaged after four weeks, probably due to oxidative stress. The observed increase in DNAsb may be a result of a suppressed antioxidant defense system, resulting in the elevation of this damage marker.

LDH has been used as a metabolic indicator of pathological organ and tissue damage [79]. In this study, significantly increased levels of LDH activity in fish exposed to P4 for one week at 50 and 100 ng/L, and for two weeks at 100 ng/L, together with the AChE activity results, indicate structural damage to the liver cells. The results obtained for mixtures also showed increased LDH activity in the liver, however the LDH activity was in the same range as single-component solutions. After one week of exposure, MIX1, MIX2, and MIX3 had a significantly increased effect on LDH activity. After two weeks, MIX1 and MIX3, and after four weeks only MIX3, resulted in a significant increase. Additionally, MIX3 had the most pronounced effects on LDH, as seen in the other markers.

The results of the biochemical markers assessed in this study indicate a synergistic dose-ratio-dependent effect of CBZ and P4 on xenobiotic metabolization enzymes and VTG in zebrafish after chronic exposure [80]. Differences in the mixture combinations revealed a non-linear response of zebrafish to the assessed mixtures. After short-term exposure to binary mixtures, oxidative stress enzyme activities were lower than expected based on single-component results. The observed marker responses to binary mixtures showed that not only time but also the proportion of the components determines the main toxicological effect of CBZ and P4. Changes of the response to oxidative stress depending on single-component solutions or binary mixtures helps to better understand the toxic effect mechanism of multiple chemical stressors. The results of VTG concentration changes also confirm the risk concerning alterations in reproductive success caused by pharmaceuticals co-appearing in surface waters [8]. This study confirms previous findings and exhibits the co-toxic effects of different chemicals, which may exceed the effects of single components, highlighting the risk to natural sustainable populations of fish and other freshwater species.

## 5. Conclusions

Chronic exposure to environmentally relevant concentrations of CBZ, P4, and their mixtures inflicted significant biochemical alterations, and to mixtures biochemical markers in zebrafish showed non-linear strengthening responses. These synergistic effects on VTG production suggests a high risk to the reproductive success of fish, if these chemicals are present simultaneously. In addition to the mixture effects on reproduction, xenobiotic metabolizing enzymes (EROD, GST) and the oxidative stress marker (DNAsb) were also significantly altered as compared to the results of single-chemical exposure after 28 days.

## Figures and Tables

**Table 1:** Composition of mixtures assessed.

	CBZ		P4		STU
	TU	µg/L	TU	ng/L	
**MIX1**	0.75	11.5	0.25	4.375	1
**MIX2**	0.5	5.75	0.5	8.75	1
**MIX3**	0.25	2.875	0.75	17.5	1

## Data Availability

We have full control of all primary data, and we agree to allow the journal to review our data if requested.

## Supplementary Materials

The following supporting information can be downloaded at: https://www.mdpi.com/article/10.3390/antiox11091776/s1, Supplement S1: Detailed description of biochemical determinations.
