# Supplementary material for: Chronic Effects of Carbamazepine, Progesterone and Their Mixtures at Environmentally Relevant Concentrations on Biochemical Markers of Zebrafish (Danio rerio)
Source: Antioxidants (Basel). 2022 Sep 8;11(9):1776. doi: 10.3390/antiox11091776 (PMC9495832; doi:10.3390/antiox11091776)
Supplement: Supplementary file 1 [file antioxidants-11-01776-s001.zip › antioxidants-1864368-supplementary.pdf]

Supplement S1. Detailed description of biochemical determinations.

Lipid peroxidation was evaluated based on the formation of malonaldehyde in tissue homogenates by the thiobarbituric acid method elaborated by [1]. A 150 µL homogenate was mixed with 300 µL of 10% TCA containing 1 mM FeSO4 and 150 µL of 0.67% thiobarbituric acid. The mixture was heated to 80 °C for 10 min, then precipitates were removed by centrifugation (10,000g for 10 s). The supernatant was subjected to fluorescence measurement at 516 excitation/600 nm emission. Blanks and standards of tetramethoxypropane were prepared in homogenization buffer. Results were expressed as µmoles of thiobarbituric acid reactants per milligram of homogenate protein.

The determination of AChE activity was carried out according to the method of Ellman et al. [2] to microplate [3]. A 96-well microplate was loaded with 3 replicates of 50 µl of homogenate supernatant and 250 µl of a solution made with 0.075 M acetylthiocholine iodide and 10 mM 5,5 dithio-bis(2-nitrobenzoic acid) in phosphate buffer (0.1 M, pH 7.2). In assay blanks samples were substituted whit phosphate buffer and electric eel acetylcholine esterase was used as positive control. Absorbance was continuously measured at 414 nm for 15 min at every min. Enzymatic activity was calculated from the slope of the absorbance curve and was expressed in Units (U) per mg of protein content (1 U being 1 µmol of substrate hydrolyzed/min).

CAT activity was measured in triplicates following the method of Aebi [4]. Decreases in the absorbance of a 50 mM H2O2 solution (ε = -0.0436 mM-1 cm-1) in 50 mM phosphate buffer (pH 7.8) and 10 µL of tissue supernatant (S12) were continuously recorded at 240 nm at 10 s intervals for 1 min. The results were expressed as U/mg protein; a unit of CAT was defined as the amount of enzyme that catalysed the dismutation of 1 mmol of H2O2/min.

GST activity was determined by the method of [5] adapted to microplate, according to the following procedure: a solution of glutathione (GSH) 100 mM in phosphate buffer (pH = 6.5), and a second solution of 60 mM 1-chloro-2,4-dinitrobenzene (CDNB, ε= 9.6 mM-1 cm-1) in ethanol was prepared just before the assay. The reaction mixture consisted of phosphate buffer, GSH solution and CDNB solution in a proportion of 4.95 ml (phosphate buffer): 0.9 ml (GSH): 0.15 ml (CDNB). In the microplate, 0.2 ml of the reaction mixture was added to 0.1 ml of the sample (S12) and the GST activity was measured immediately at every 20 seconds, at 340 nm, during the first 5 minutes. GST from equine liver was used as positive control. Enzymatic activity was calculated from the slope of the absorbance curve and was expressed in Units (U) per mg of protein content (1 U being 1 µmol of substrate hydrolyzed/min).

Glutathione peroxide activities were measured according to Paglia and Valentine [6] modified by Lawrence and Burk [7] and adapted to a 96-well microplate [8]. The reaction mixture contained 30 µL sample (S12) 100 mM phosphate buffer (pH 7.5), 2 mM GSH, 2 U glutathione reductase, 0.12 mM NADPH, sodium azide (0.5 mM), 0.2 mM H2O2 or 3 mM cumene hydroperoxide (CHP). GPX activity was monitored by following the decrease in NADPH concentration (at 340 nm), which is consumed during the generation of GSH from oxidized glutathione (ε=6.2 cm−1 M−1), using H2O2 (Se dependent activity), or cumene hydroperoxide (total GPX) as substrate. GPx activity was expressed as U per mg of protein. (a U corresponding to 1 µM NADPH hydrolysed/min).

GR activity was also measured by the decrease of NADPH at 340 nm for 1 min and expressed as µM/mg prot./min according to Carlberg and Mannervik [9]. The reaction medium contained 100 mM phosphate buffer (pH=7.4), 0.1 mM NADPH and 30 µL supernatant (S12).

LDH activity was measured according to the methodology described by Vassault [10], adapted to microplate by Diamantino et al. [11]. In the liver 25 µL of buffer Tris-NaCl (0.1 M, pH 7.2) and 125 µL

of nicotinamide adenine dinucleotide reduced (NADH) (300 μM) were added to 20 μL of pyruvate (4.5 mM). Reading was performed at 340 nm at intervals of 40 s for 5 min, following a decrease of absorbance resulting from oxidation of NADH.

EROD activity was determined in the 12,000g microsomal fraction according to Burke and Mayer [12]. Subsamples of tissue homogenates were centrifuged at 12,000g for 30 min at 4 °C. Fifty microliters of supernatant were incubated at 30 °C for 60 min in a final volume of 200 μL containing 100 mM phosphate buffer, pH 7.4, 100 μM reduced NADPH and 10 μM 7-ethoxyresorufin. The reaction was started by the addition of NADPH, and stopped by the addition of 100 μL of 0.5 M NaOH. The resultant 7-hydroxyresorufin was determined by fluorometry at 520 nm excitation and 590 nm emission wavelengths. Calibration was performed with serial dilutions of 7- hydroxyresorufin. Results were expressed as total protein.

DNA strand breaks were quantified by an adaptation of the alkaline precipitation assay of Olive [13]. A 25 μL tissue homogenate was mixed with 200 μL of 2% SDS containing 10 mM EDTA, 10 mM Trisbase and 40 mM NaOH and shaken for 1 min. Two hundred microliter of 0.12 M KCl was added, the mixture was further heated at 60 °C for 10 min, mixed by inversion and cooled at 4 °C for 30 min then, centrifuged at 8000g for 5 min at 4 °C. Fifty microliter of supernatant was added to 150 μL of Hoechst dye (1 μg mL−1 , in buffer containing 0.4 M NaCl, 4 mM sodium cholate and 0.1 M Tris-acetate, pH 8.5–9 and mixed for 5 min on a plane shaker). Fluorescence was measured at 360 excitation/450 nm emission wavelengths. Blanks contained identical constituents, with 25 μL Hepes buffer replacing the tissue homogenate. Salmon sperm DNA standard (Sigma) was used for DNA calibration and the results were expressed as DNA_sb μg mg−1 protein.

Vitellogenin-like proteins (Vtg) were determined in the 12,000g microsomal fraction following the alkali-labile phosphate (ALP) method developed by Blaise et al. [14]. Two hundred microliter of sample homogenate was mixed with 54 μL acetone (35% final concentration) for 10 min and centrifuged at 10,000g for 5 min. The retained pellet was then dissolved in 50 μL 1 M NaOH and mixed for 30 min at 60 °C. The total phosphate was then determined by the colorimetric phosphomolybdenum method developed by Stanton [15]. To a 20 μL sample 125 μL H2O, 5 μL 100% TCA, 25 μL of molybdate reactive and 25 μL ascorbate 1% were added, mixed for 10 min and the absorbance was read at 815 nm and 444 nm. Rainbow trout vitellogenin was used for calibration and aliquots of NaOH (1 M) were used as blanks. Vtg levels were expressed as μmoles of ALP per milligram of protein.

Total SOD activity was measured using the xanthine oxidase/cytochrome c method proposed by Crapo [16] in S12 fraction. Cytochrome c reduction by superoxide anions generated by the xanthine oxidase/hypoxanthine reaction was detected at 550 nm at room temperature. Enzyme activity was expressed as U/mg protein; a unit of SOD was defined as the amount of sample producing 50% inhibition under the assay conditions. The reaction mixture contained 46.5 mM KH2PO4/K2HPO4 (pH 8.6), 0.1mM EDTA, 195 mM hypoxanthine, 16 mM cytochrome c, and 2.5 mU xanthine oxidase. The enzymatic activity was calculated from the slope of the absorbance curve and was expressed in Units (U) per mg of protein (1 U causing 50 % inhibition of the rate of cytochrome c reduction).

The values of each biomarker were normalized against the protein content of either the whole homogenate or supernatant [17].

1. Wills, E.D. Evaluation of Lipid Peroxidation in Lipids and Biological Membranes. In *Biochemical Toxicology: A Practical Approach*; Snell, K., Mullock, B., Ed.; IRL Press: Washington, DC, 1987; pp. 127–152.

2. Ellman, G.L.; Courtney, K.D.; Andres, V.; Featherstone, R.M. A New and Rapid Colorimetric Determination of Acetylcholinesterase Activity. *Biochemical Pharmacology* **1961**, doi:10.1016/0006-2952(61)90145-9.

3. Guilhermino, L.; Lopes, M.C.; Carvalho, A.P.; Soares, A.M.V.M. Inhibition of Acetylcholinesterase Activity as Effect Criterion in Acute Tests with Juvenile Daphnia Magna. *Chemosphere* **1996**, doi:10.1016/0045-6535(95)00360-6.

4. Aebi, H. [13] Catalase in Vitro. *Methods in Enzymology* **1984**, *105*, 121–126, doi:10.1016/S0076-6879(84)05016-3.

5. Habig, W.H.; Pabst, M.J.; Jakoby, W.B. Glutathione S Transferases. The First Enzymatic Step in Mercapturic Acid Formation. *Journal of Biological Chemistry* **1974**.

6. Paglia, D.E.; Valentine, W.N. Studies on the Quantitative and Qualitative Characterization of Erythrocyte Glutathione Peroxidase. *The Journal of Laboratory and Clinical Medicine* **1967**, doi:10.5555/uri:pii:0022214367900765.

7. Lawrence, R.A.; Burk, R.F. Glutathione Peroxidase Activity in Selenium-Deficient Rat Liver. *Biochemical and Biophysical Research Communications* **1976**, *71*, 952–958, doi:10.1016/0006-291X(76)90747-6.

8. Faria, M.; Carrasco, L.; Diez, S.; Riva, M.C.; Bayona, J.M.; Barata, C. Multi-Biomarker Responses in the Freshwater Mussel Dreissena Polymorpha Exposed to Polychlorobiphenyls and Metals. *Comparative Biochemistry and Physiology - C Toxicology and Pharmacology* **2009**, *149*, 281–288, doi:10.1016/j.cbpc.2008.07.012.

9. Carlberg, I.; Mannervik, B. Purification and Characterization of the Flavoenzyme Glutathione Reductase from Rat Liver. *Journal of Biological Chemistry* **1975**.

10. Vassault, A. Lactate Dehydrogenase. In *Methods of enzymatic analysis III*; Bergmeye, H.U., Bergmeyer, J., Gra, I.M., Eds.; Verlag Chemie, 1983; pp. 118–126.

11. Diamantino, T.C.; Almeida, E.; Soares, A.M.V.M.; Guilhermino, L. Lactate Dehydrogenase Activity as an Effect Criterion in Toxicity Tests with Daphnia Magna Straus. *Chemosphere* **2001**, *45*, 553–560, doi:10.1016/S0045-6535(01)00029-7.

12. Burke, M.D.; Mayer, R.T. Ethoxyresorufin: Direct Fluorimetric Assay of a Microsomal O Dealkylation Which Is Preferentially Inducible by 3 Methylcholanthrene. *Drug Metabolism and Disposition* **1974**.

13. Olive, P.L. DNA Precipitation Assay: A Rapid and Simple Method for Detecting DNA Damage in Mammalian Cells. *Environmental and Molecular Mutagenesis* **1988**, *11*, 487–495, doi:10.1002/em.2850110409.

14. Blaise, C.; Gagné, F.; Pellerin, J.; Hansen, P.D. Determination of Vitellogenin-like Properties in Mya Arenaria Hemolymph (Saguenay Fjord, Canada): A Potential Biomarker for Endocrine Disruption. *Environmental Toxicology* **1999**, *14*, 455–465, doi:10.1002/(SICI)1522-7278(199912)14:5<455::AID-TOX2>3.0.CO;2-8.

15. Stanton, M.G. Colorimetric Determination of Inorganic Phosphate in the Presence of Biological Material and Adenosine Triphosphate. *Analytical Biochemistry* **1968**, *22*, 27–34, doi:10.1016/0003-2697(68)90255-8.

16. Crapo, J.D.; McCord, J.M.; Fridovich, I. [41] Preparation and Assay of Superioxide Dismutases. In *Methods in Enzymology*; 1978; Vol. 53, pp. 382–393.

17. Bradford, M. A Rapid and Sensitive Method for the Quantitation of Microgram Quantities of Protein Utilizing the Principle of Protein-Dye Binding. *Analytical Biochemistry* **1976**, *72*, 248–254, doi:10.1006/abio.1976.9999.
